# Female With Hypertensive Emergency Later Found to Have ANCA-Associated Vasculitis: A Case Report

**DOI:** 10.7759/cureus.66835

**Published:** 2024-08-14

**Authors:** Alaa Aldookhi, Ahmed Almagazzachi, Bushra Ghafoor, Mowyad Khalid

**Affiliations:** 1 Internal Medicine, Capital Health Regional Medical Center, Trenton, USA; 2 Internal Medicine, Capital Health System, Trenton, USA; 3 Rheumatology, Capital Health System, Trenton, USA

**Keywords:** anca-associated vasculitis, petechiae rash, diffuse alveolar hemorrhage, rapidly progressive glomerulonephritis (rpgn), hypertension emergency

## Abstract

Granulomatosis with polyangiitis (GPA) is a form of ANCA-associated vasculitis characterized by necrotizing vasculitis affecting small blood vessels. The clinical presentation varies based on organ involvement, commonly affecting the upper and lower respiratory tracts and kidneys. Typical GPA presents as recurrent sinus infection, otitis media, dyspnea, chest pain, and glomerulonephritis, which can present as hematuria, proteinuria, and elevated serum creatinine. ANCA tests positive in the majority of cases. Treatment strategies involve induction of remission and maintenance therapy.

We report a case of a 48-year-old female presenting with a hypertensive emergency, a rarely reported manifestation of GPA. She initially presented with severe headache and cough, with systolic blood pressure exceeding 220 mmHg, necessitating hospital admission. The initial workup revealed elevated serum creatinine and CT chest findings suggestive of multi-lobar pneumonia, for which she received antibiotic treatment. Despite aggressive antihypertensive therapy, her blood pressure remained refractory, and she developed hematuria and anemia, requiring a blood transfusion. Further evaluation revealed a history of joint pain, recurrent sinus infections, and a pruritic skin rash, prompting suspicion of vasculitis. Further work-up included elevated erythrocyte sedimentation rate (ESR), normal IgE, absence of eosinophilia, and positive PR3 antibodies and c-ANCA. Prompted by clinical suspicion, treatment with steroids was initiated, and a kidney biopsy confirmed acute necrotizing pauci-immune glomerulonephritis consistent with GPA. Subsequently, rituximab therapy was initiated, resulting in significant improvement in her clinical symptoms and blood pressure, and the patient was successfully discharged home.

This case highlights a rare presentation of GPA as a hypertensive emergency, possibly linked to renal involvement in the form of glomerulonephritis. Pulmonary manifestations mimicking infections posed diagnostic challenges. Cutaneous findings potentially associated with increased joint and renal involvement underscore the clinical complexity of GPA. The unusual presentation of hypertensive emergency in young patients underscores the need for heightened awareness of this potential manifestation in GPA. Early recognition and aggressive immunosuppressive therapy are crucial to mitigate irreversible renal damage in such atypical presentations.

## Introduction

ANCA-associated vasculitis represents a group of disorders including granulomatosis with polyangiitis (GPA), renal limited vasculitis, microscopic polyangiitis (MPA), and eosinophilic granulomatosis with polyangiitis (EGPA) [1]. The prevalence of GPA is 2.3-146 cases per million persons and the incidence of about 0.4-11.9 cases per million a year [2].

As small blood vessels necrotizing vasculitis, both GPA and MPA present different manifestations according to the organ involvement and disease severity, but they most commonly affect the upper respiratory tract, lower respiratory tract, and kidneys. They can present as life-threatening diseases or have less severe or atypical presentations. ENT manifestations are more common with GPA than with MPA, and the manifestations include recurrent sinusitis, otitis media, and persistent rhinorrhea [3]. Pulmonary manifestations include wheezing, chronic cough, chest pain, and dyspnea. Radiographic findings include patchy or diffuse pulmonary infiltrate and hilar lymphadenopathy [4]. Kidney manifestations include glomerulonephritis and the symptoms include asymptomatic hematuria, increased serum creatinine, and a variable degree of proteinuria [5]. Cutaneous manifestations include cutaneous vasculitis, purpura or possible focal necrosis, and ulceration [6]. Other manifestations include eye involvement like conjunctivitis, episcleritis/scleritis, and neurological manifestations like mononeuritis multiplex [7]. EGPA differs in presentation from GPA and MPA by including asthma and eosinophilia, but can also affect other organs like skin, peripheral nerves, and lungs [1]. ANCA tests positive in 82-94% of cases of GPA and MPA and depends also on the severity of cases [8], while it's positive in 30-40% of cases of EGPA [9]. 

Renovascular diseases are the most common cause of secondary hypertension, accounting for 1-5% of all causes of hypertension in the general population and 5.4% of secondary hypertension in young people. Of renovascular hypertension cases, 90% are caused by atherosclerotic renal artery stenosis, 9% are caused by fibromuscular dysplasia, and miscellaneous causes including vasculitis cause 1% of cases [10]. The GPA presentation as a hypertensive emergency is rarely reported in the literature. We present the case of a young lady with no significant history. Her main complaint was headache and high blood pressure refractory to antihypertensive medications. Although she reported some shortness of breath, it was not the main complaint.

## Case presentation

A 48-year-old female with no past medical history (she had not visited any doctor for years) went to urgent care for a headache and cough. During that visit, her blood pressure was very high, and she was transferred to the emergency department for evaluation and the treatment of new-onset severe hypertension. In the ER, the patient was found to have a hypertensive emergency. Her systolic blood pressure was above 220 and her labs were remarkable for elevated serum creatinine (CR) of 3.4, with an unknown baseline, and the patient had a cough and shortness of breath. So, she was admitted to the hospital for further evaluation and management of hypertensive emergency. 

**Figure 1:**
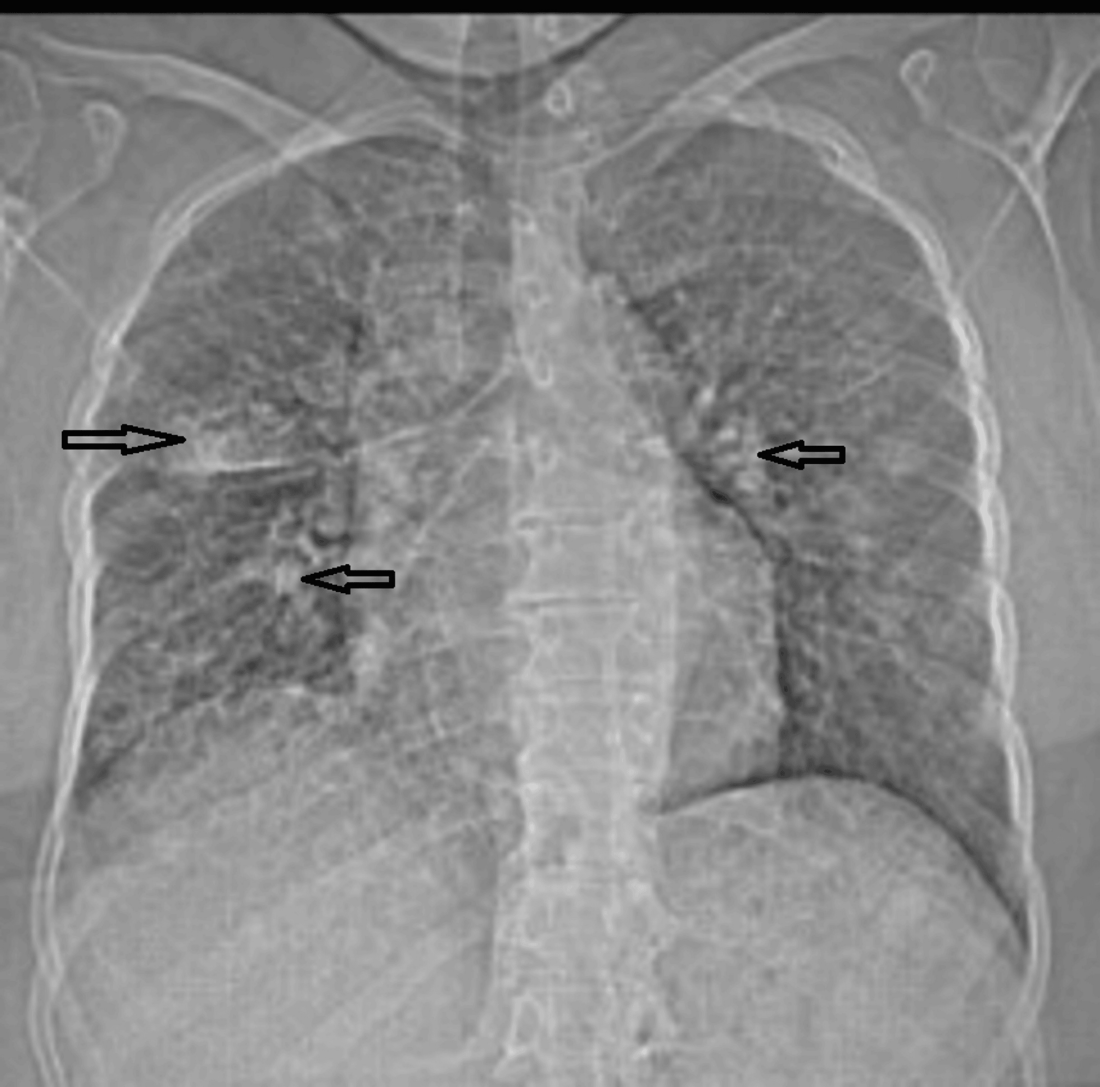
Chest X-ray shows bilateral diffuse ground glass opacities and infiltrates (arrows), suspicious for pneumonia.

**Figure 2:**
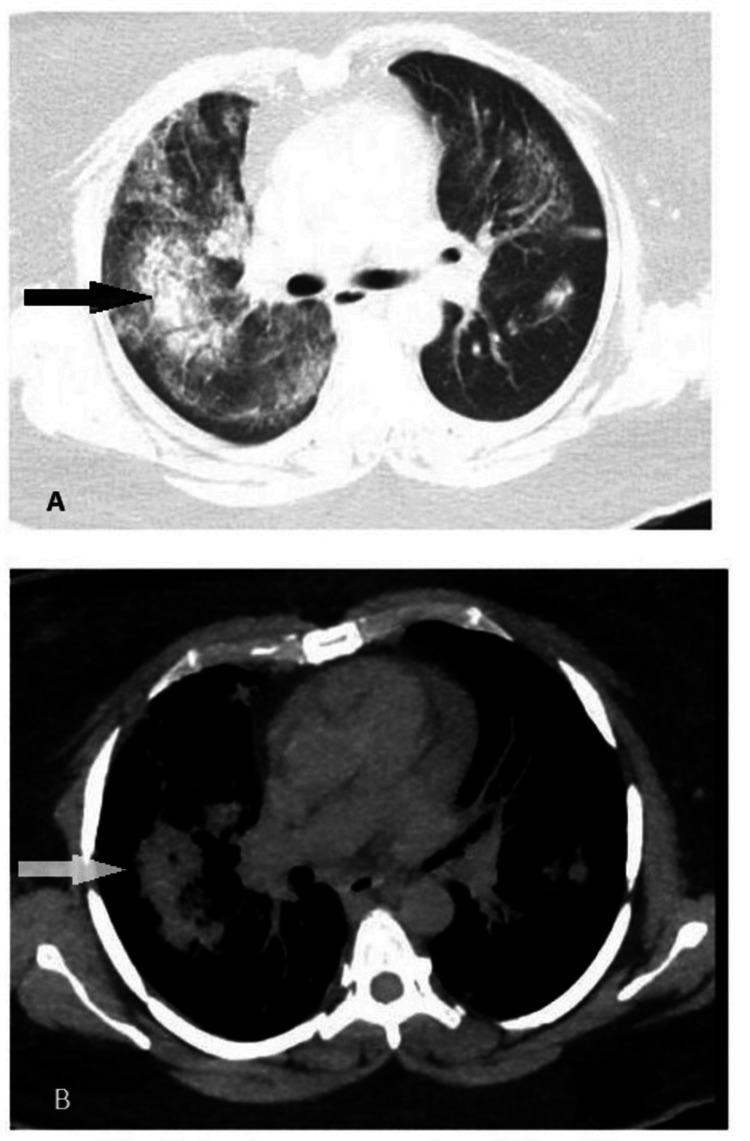
Different images (A and B) of the CT scan of the chest. White and black arrows point toward the areas of diffuse alveolar hemorrhage, which are ground glass opacities.

**Figure 3:**
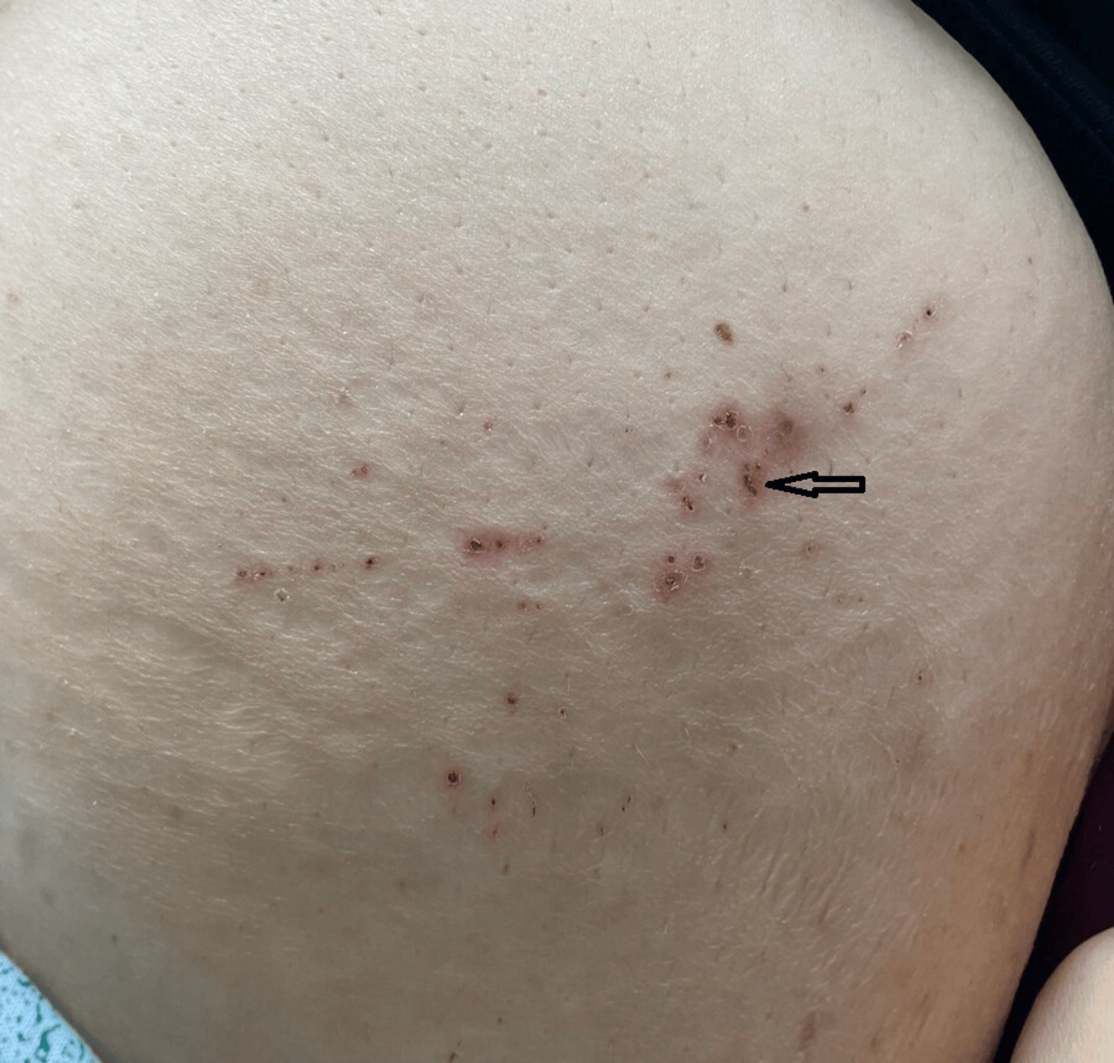
Arrow points to one of multiple pruritic pinpoint petechial skin rashes noted on the anterior surface of the abdominal wall.

During hospitalization, the patient’s chest X-ray and CT scan of the chest showed a bilateral patchy infiltrate, mainly on the right side, which was read as multi-lobar pneumonia (Figures 1, 2). So, the patient was started on antibiotics. She was also treated for a hypertensive emergency with more than two oral medications, but her blood pressure was very high and she was resistant to the medications. The patient also developed hematuria during hospitalization for an unknown reason and had a normal kidney and bladder ultrasound. Her hemoglobin dropped to 6.2 and she received one unit of packed RBCs. In history taking, the patient reported joint pain on her hand and shoulders with pinpoint superficial pruritic skin rash (Figure 3) and also mentioned she had recurrent sinus infections in the past. Labs were remarkable for elevated erythrocyte sedimentation rate (ESR) (55, N<30), urinary protein-to-CR (UPC) ratio (4.37, N<0.2), uric acid (UA) with blood, antinuclear antibody (ANA) (1:160), anti-PR-3 antibodies (>8, N<1), and c-ANCA (1:320, N<1:20). C-reactive protein (CRP) was 2, and rheumatoid factor (RF) was 105 (N<14).

Because of the recurrent sinopulmonary infections, lung involvement on the CT scan, glomerulonephritis (CR peaked at 3.4), and inflammatory arthritis on her hands and shoulders, the patient was treated for a possible ANCA vasculitis of the GPA type. it was unlikely to be EGPA with the absence of eosinophilia, normal Ig E, and PR3 positivity. Nephrology was consulted for kidney biopsy and rheumatology was involved in her care. The patient was started on pulse steroids, which included methylprednisolone IV 1000 mg daily for three days then she was transitioned to prednisone oral tab tapering. She noticed an improvement in her breathing and inflammatory arthritis. After that, the kidney biopsy confirmed the diagnosis of ANCA vasculitis, which had taken the form of acute necrotizing glomerulonephritis of the pauci-immune type. The patient was then started with rituximab therapy and was then discharged home.

## Discussion

GPA, formerly known as Wegener's granulomatosis, is a rare and complex autoimmune disease characterized by necrotizing granulomatous inflammation and vasculitis of small to medium-sized vessels. This case report illustrates an unusual presentation of GPA in a 48-year-old female, whose clinical course was marked by hypertensive emergency, multi-lobar pneumonia, glomerulonephritis, and inflammatory arthritis.

The presentation of hypertensive emergency in GPA is exceptionally rare, making this case particularly noteworthy. Hypertensive emergencies are typically related to secondary causes, and in this case, the severe hypertension observed upon initial evaluation, with systolic blood pressure exceeding 220 mmHg, can be attributed to underlying renal pathology. According to a study by Falk et al. (1990), renal involvement in GPA often leads to glomerulonephritis, which can cause significant hypertension due to impaired renal function [11]. The resistant nature of the hypertension, despite multiple antihypertensive medications, underscores the severity of the renal involvement and necessitates aggressive management.

Pulmonary manifestations are common in GPA and can often mimic infectious processes, leading to diagnostic challenges. Our patient’s cough and shortness of breath, coupled with CT findings of bilateral patchy infiltrates predominantly on the right side, were initially interpreted as multi-lobar pneumonia. The literature indicates that pulmonary involvement in GPA can present as infiltrates, nodules, or even diffuse alveolar hemorrhage [12]. Distinguishing between infectious and inflammatory etiologies is crucial, as highlighted by the recurrent sinopulmonary infections seen in this case, which align with GPA's clinical spectrum.

Renal impairment is a severe and common complication of GPA, often presenting as rapidly progressive glomerulonephritis (RPGN). In this case, significant renal involvement was indicated by elevated serum CR levels (3.4 mg/dL). A kidney biopsy confirmed pauci-immune necrotizing glomerulonephritis, a hallmark of GPA. According to an observational study published in 2021, 35% of hospitalized patients with GPA had renal involvement [13]. Early and aggressive immunosuppressive therapy is essential to prevent irreversible renal damage, as demonstrated in our patient’s management.

Joint pain and inflammatory arthritis, particularly in the hands and shoulders, are frequent extra-pulmonary manifestations of GPA. This patient also reported a pruritic skin rash, which, although less common, can present in various forms such as rashes and purpura. This is consistent with findings by Frances et al. [14], who investigated the possible link between cutaneous manifestations and systemic involvement. They concluded that cutaneous manifestations might be linked to an increased frequency of articular and renal involvement. The presence of these symptoms in our patient further supports the multi-system involvement typical of GPA.

Laboratory findings play a critical role in diagnosing GPA. In this case, elevated ESR (55 mm/hr), UPC ratio (4.37 g/g), positive ANA (1:160), markedly high anti-PR3 antibodies (>8), and a c-ANCA titer of 1:320 were highly suggestive of GPA. The specificity of anti-PR3 and c-ANCA for GPA has been well-documented in the literature [15], making these markers indispensable in the diagnostic process.

The treatment of ANCA-associated vasculitis includes two phases: induction of remission and maintenance phase. Several trials were used to assess the efficacy of rituximab plus steroids in both phases (Table 1) [16]. Rituximab is preferable due to the side effects and toxicity of cyclophosphamide and the relapsing feature of ANCA-associated vasculitis. The ADVOCATE, multicenter, phase III, randomized, double-blind, placebo-controlled trial showed avacopan, a C5a receptor antagonist, to be not inferior to prednisone in the maintenance phase [17]. FDA approval indications for new ANCA-associated vasculitis medications are summarised in Table 2 [16].

**Table 1:** Trials were used to assess the efficacy of ANCA-associated vasculitis (AAV) drugs in both induction of remission and maintenance phase. RTX: rituximab; RAVE: Rituximab in ANCA-Associated Vasculitis; RITUXVAS: Rituximab Versus Cyclophosphamide in ANCA-Associated Vasculitis; MAINRITSAN: Maintenance of Remission using Rituximab in Systemic ANCA-associated Vasculitis; REOVAS: Rituximab in Eosinophilic Granulomatosis With Polyangiitis; CLASSIC: Conservative versus Liberal Approach to Fluid Therapy of Septic Shock in Intensive Care; ADVOCATE: Avacopan Development in Vasculitis to Obtain Corticosteroid Elimination and Therapeutic Efficacy; CLEAR: Cholesterol Lowering via Bempedoic acid, an ACL-Inhibiting Regimen; MIRRA: Mepolizumab in Relapsing or Refractory EGPA; CYC: cyclophosphamide; AZA: azathioprine; CS: corticosteroids; EGPA: eosinophilic granulomatosis with polyangiitis Source: [17]

Target	Drug	Trial	Primary endpoint results
B cells	RTX	RAVE	Non-inferiority to oral CYC for remission induction, superior for relapsing or PR3-ANCA patients
B cells	RTX	RITUXVAS	Non-inferiority to CYC in pulses for remission induction
B cells	RTX	MAINRITSAN	Superiority to AZA for the maintenance of remission
B cells	RTX	MAINRITSAN 2	No difference between standard and customized infusion based on B-cell count for relapse rate
B cells	RTX	REOVAS	Non-inferiority to conventional therapy for remission (CYC/CS) in EGPA
C5aR	Avacopan	CLASSIC	Safe and effective at day 85
C5aR	Avacopan	ADVOCATE	Non-inferiority to CS for remission induction
C5aR	Avacopan	CLEAR	Non-inferiority to CS for remission induction
IL-5	Mepolizumab	MIRRA	Non-inferiority to placebo for relapsing or refractory EPGA

**Table 2:** FDA-approved indications for new ANCA-associated vasculitis (AAV) drugs. GPA: granulomatosis with polyangiitis; MPA: microscopic polyangiitis; EGPA: eosinophilic granulomatosis with polyangiitis Source: [18]

^Drug^	^AAV^	^Specification^
_Rituximab_	_GPA and MPA_	_In adult and pediatric patients two years of age and older in combination with glucocorticoid_
_Avacopan_	_GPA and MPA_	_As an adjunctive treatment for adult patients with severely active GPA and MPA in combination with standard therapy_
_Mepolizumab_	_EGPA_	_Adult patients with EGPA_

The management of this case involved high-dose pulse steroids (1000 mg daily for three days), followed by a tapering dose of oral prednisone, leading to a significant improvement in respiratory and joint symptoms. The initiation of rituximab, an anti-CD20 monoclonal antibody, was crucial. Rituximab has been shown to be effective in inducing remission in GPA, particularly in patients with severe renal involvement [18]. The patient's clinical improvement following rituximab therapy underscores its efficacy in managing refractory cases of GPA.

This case aligns with findings from various case reports and studies on GPA. For example, the presentation of hypertensive emergency, although rare, can be attributed to severe renal involvement. Pulmonary infiltrates and renal impairment are consistent with the typical manifestations of GPA [19]. Additionally, inflammatory arthritis and skin manifestations are frequent extrapulmonary features [20].

## Conclusions

This case highlights the complexity and diagnostic challenges associated with GPA, emphasising the necessity for a multidisciplinary approach in managing such patients. Early recognition and aggressive immunosuppressive therapy are essential to prevent severe organ damage and improve patient outcomes. The unusual presentation of hypertensive emergency underscores the need for heightened awareness of this potential manifestation in GPA.
